# 'What did the doctor say again?' - Patient Expectations Prior To Cardiac Surgery

**DOI:** 10.1186/1749-8090-10-S1-A136

**Published:** 2015-12-16

**Authors:** Michael MacPherson, Gwyn Beattie, John Butler, Nawwar Al-Attar

**Affiliations:** 1 Cardiothoracic Department, Golden Jubilee National Hospital, Greater Glasgow and Clyde, G81 4HX, UK

## Background/Introduction

Gaining informed consent is a key element of safe and transparent patient care. Although cardiac surgery is considered gold standard in many cases of complex cardiac disease, it is associated with significant complications including mortality.

## Aims/Objectives

To assess patient expectations prior to cardiac surgery, including awareness of complications.

## Method

Consecutive cases were interviewed over a two week period within a regional cardiothoracic centre in Scotland. Thirty patients were interviewed by the lead author 24 hours prior to procedure to ensure consistency.

## Results

There were 28 elective and 2 emergency cases. All patients had read the pre-cardiac surgery information booklet and signed the centre's standardised consent form detailing both major and minor complications. 2 patients (7%) gave the wrong name of procedure and 5 (17%) patients were unable to state procedure indication. When invited to list complications, 5 patients (17%) mentioned stroke, however, 22 (73%) were unaware of this potential risk when asked directly. 9 patients (30%) were able to list 3 or more complications. Most frequently recalled were mortality, bleeding and need for repeat operation. 23 (77%) correctly quoted percentage mortality risk. Interestingly, 5 (17%) patients 'did not want to know' and 2 (7%) patients were unable to recall any complications. When considering duration of hospital stay, 3 (10%) patients expected 3-5 days post operatively. 22 (73%) expected a 5-7 day stay whilst 5 (17%) anticipated greater than 7 days. When asked 'How well informed do you feel?', the mean score was 8.5/10.

## Discussion/Conclusion

This project offered valuable insight into patient expectations and knowledge of complications prior to cardiac surgery. The results show that the majority of patients were unable to recall more than 3 complications. Of note, some patients stated that they did not want to know nor discuss complications, which may reflect pre-operative mentality rather than disregard for risk. Important reflective points include emphasis on the dynamic nature of the consent process and ensuring patient understanding throughout. Recommendations have included patient information booklets being readily available at the bedside and encouraging patients and families to seek clarification, even after the consent form has been signed at pre-assessment clinic

